# Supplementary material for: Safety of the Breast Cancer Adjuvant Radiotherapy in Ataxia–Telangiectasia Mutated Variant Carriers
Source: Cancers (Basel). 2024 Apr 5;16(7):1417. doi: 10.3390/cancers16071417 (PMC11010818; doi:10.3390/cancers16071417)
Supplement: Supplementary file 1 [file cancers-16-01417-s001.zip › cancers-2925469-supplementary.pdf]

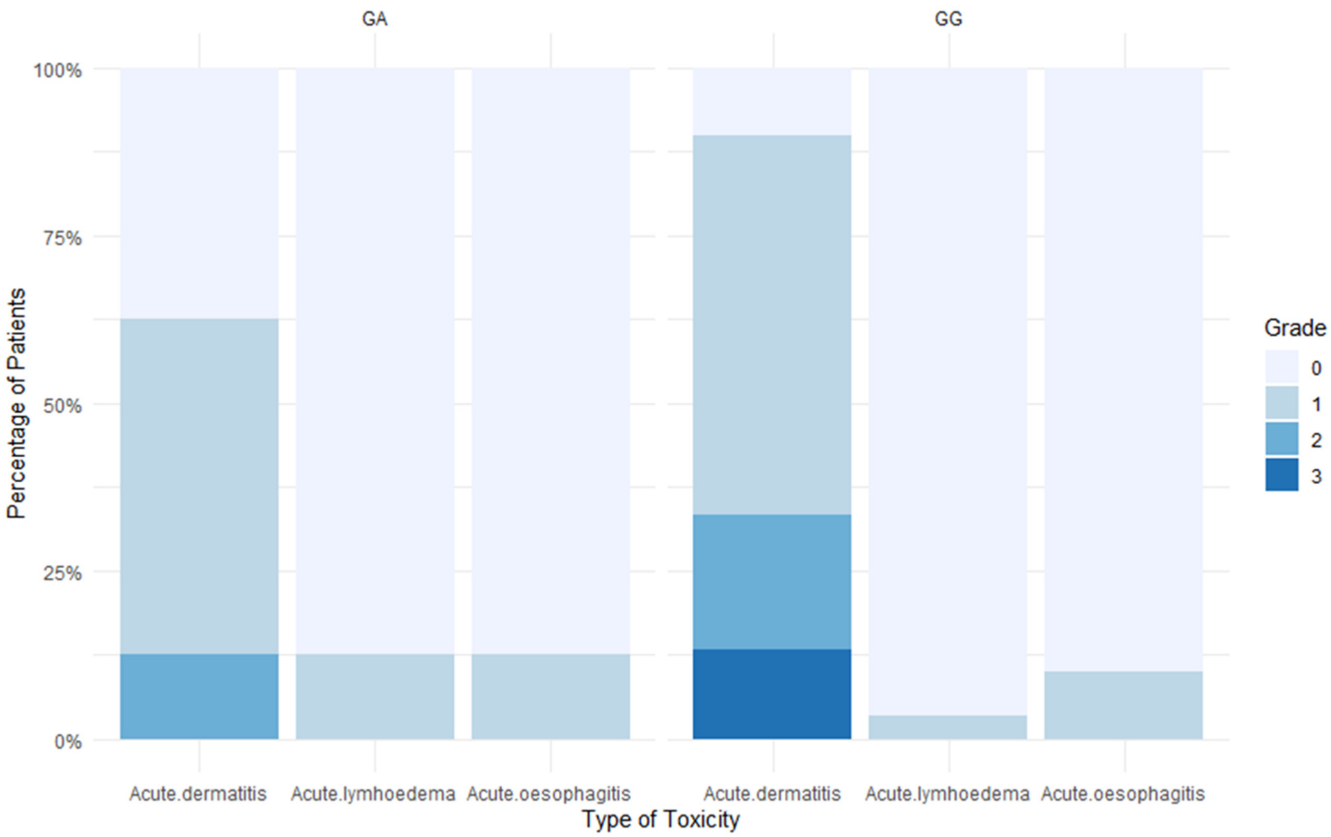

Figure S1. Acute toxicities in carriers and noncarriers of allele A of SNP rs1801516.

Abbreviations: GA: patients with genotype GA for SNP rs1801516, GG: patients with genotype GG for SNP rs1801516.

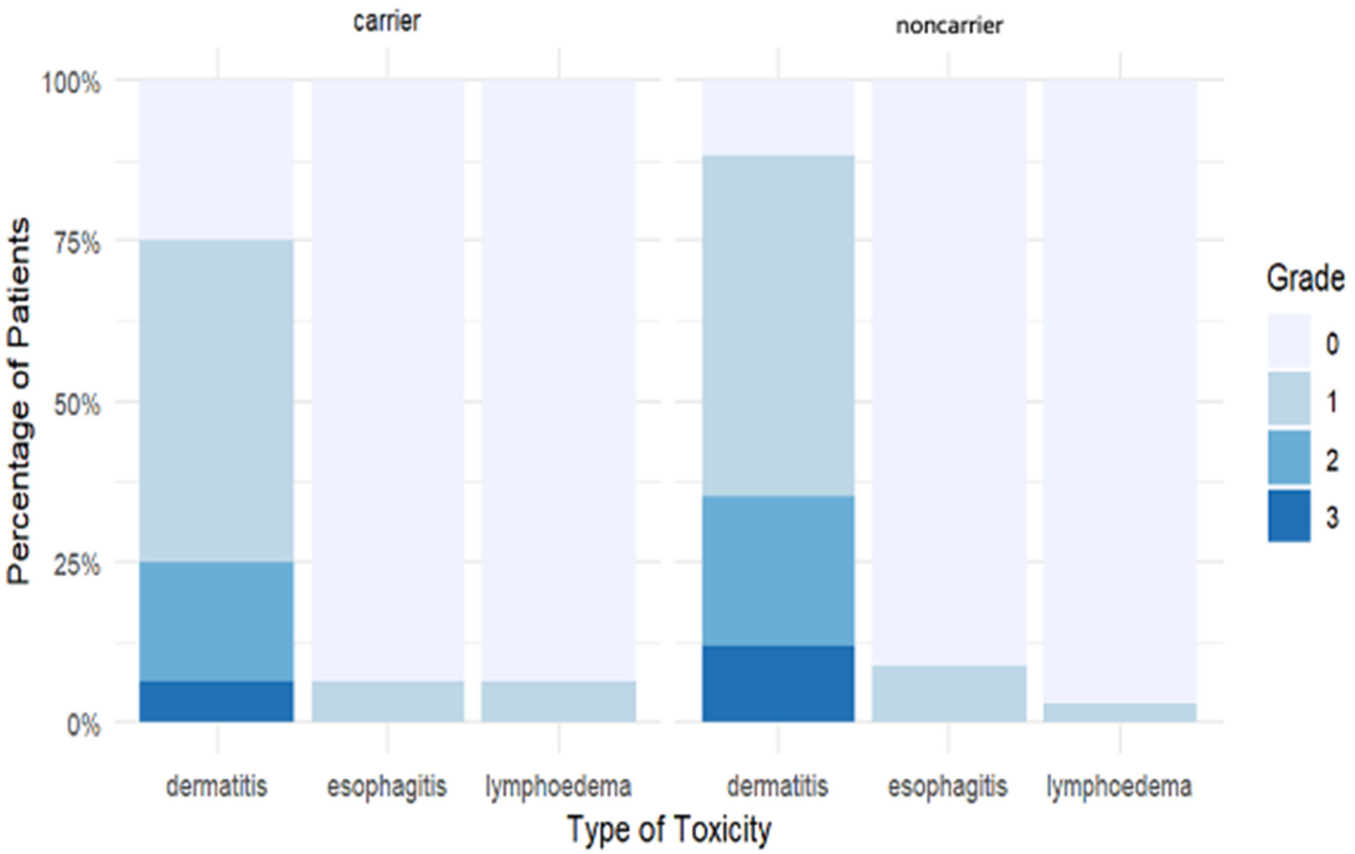

Figure S2. Acute toxicities between in carriers and noncarriers of a rare monoallelic *ATM* PV or predicted PV and/or of allele A of SNP rs1801516.

Abbreviations: Carriers : patients with rare monoallelic *ATM* PV or predicted PV and/or of allele A of SNP rs1801516. Non-carrier: patient with no *ATM* variant.

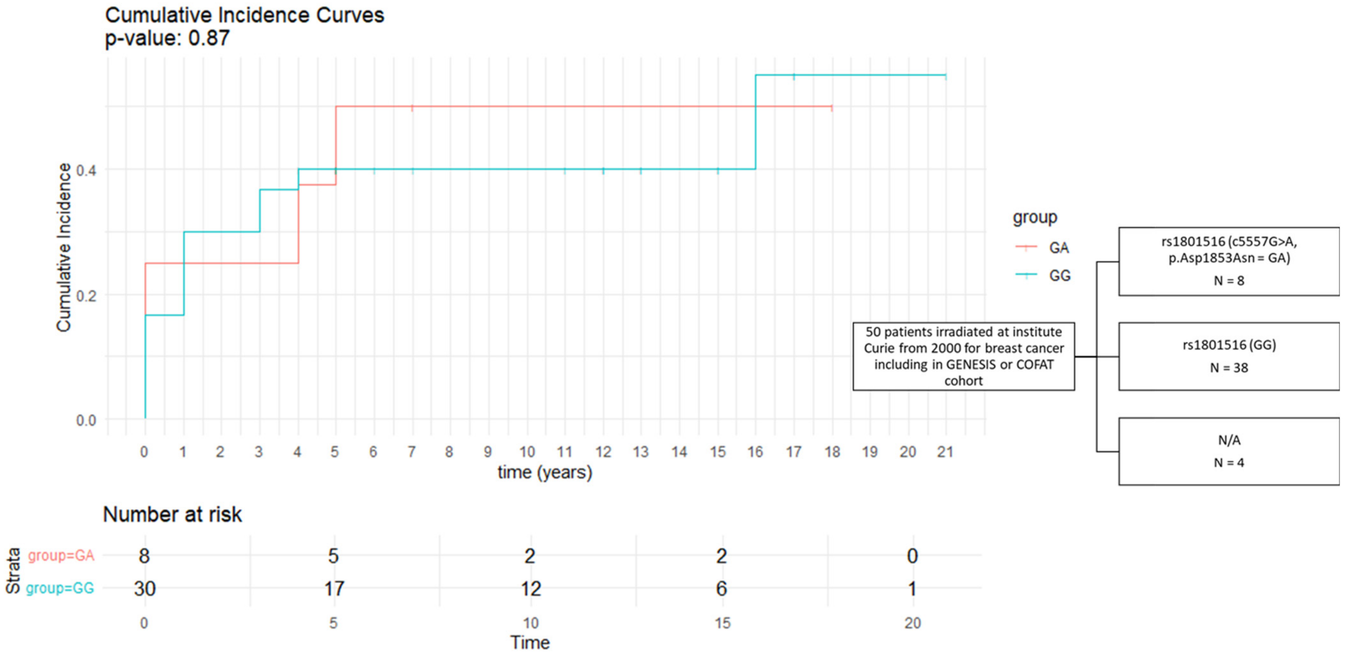

Figure S3. Incidence curve of late subcutaneous fibrosis according to genotype for SNP rs1801516.

Abbreviations: GA: patients with genotype rs1801516-GA, GG: patients with genotype rs1801516-GG, N/A: Not Available

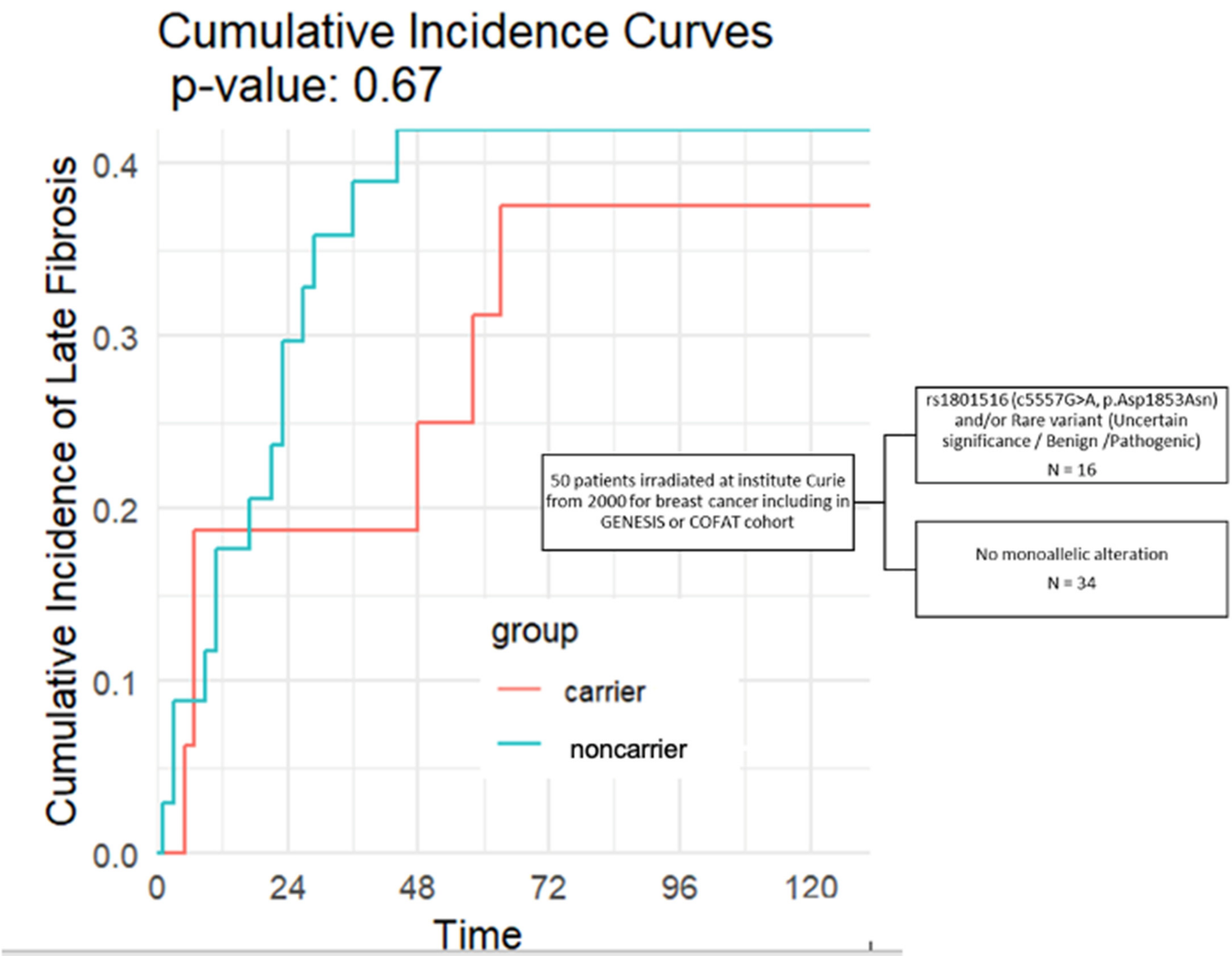

Figure S4. Incidence curve of late subcutaneous fibrosis in carriers and noncarriers of a rare monoallelic *ATM* PV or predicted PV and/or of allele A of SNP rs1801516.

Abbreviations: Carriers: patients with a rare monoallelic *ATM* PV or predicted PV and/or of allele A of SNP rs1801516. Non-carrier: patients with no *ATM* variant.
